# Genomic Regions Associated with Wool, Growth and Reproduction Traits in Uruguayan Merino Sheep

**DOI:** 10.3390/genes14010167

**Published:** 2023-01-07

**Authors:** Zully Ramos, Dorian J. Garrick, Hugh T. Blair, Brenda Vera, Gabriel Ciappesoni, Paul R. Kenyon

**Affiliations:** 1 School of Agriculture and Environment, Massey University, Palmerston North 4410, New Zealand; 2 National Research Program on Meat and Wool Production, Instituto Nacional de Investigación Agropecuaria, INIA Las Brujas, Ruta 48 Km 10, Canelones 90100, Uruguay

**Keywords:** GWAS, gene, sheep, fiber diameter, body condition score, reproduction

## Abstract

The aim of this study was to identify genomic regions and genes associated with the fiber diameter (FD), clean fleece weight (CFW), live weight (LW), body condition score (BCS), pregnancy rate (PR) and lambing potential (LP) of Uruguayan Merino sheep. Phenotypic records of approximately 2000 mixed-age ewes were obtained from a Merino nucleus flock. Genome-wide association studies were performed utilizing single-step Bayesian analysis. For wool traits, a total of 35 genomic windows surpassed the significance threshold (PVE ≥ 0.25%). The proportion of the total additive genetic variance explained by those windows was 4.85 and 9.06% for FD and CFW, respectively. There were 42 windows significantly associated with LWM, which collectively explained 43.2% of the additive genetic variance. For BCS, 22 relevant windows accounted for more than 40% of the additive genetic variance, whereas for the reproduction traits, 53 genomic windows (24 and 29 for PR and LP, respectively) reached the suggestive threshold of 0.25% of the PVE. Within the top 10 windows for each trait, we identified several genes showing potential associations with the wool (e.g., IGF-1, TGFB2R, PRKCA), live weight (e.g., CAST, LAP3, MED28, HERC6), body condition score (e.g., CDH10, TMC2, SIRPA, CPXM1) or reproduction traits (e.g., ADCY1, LEPR, GHR, LPAR2) of the mixed-age ewes.

## 1. Introduction

The genetic improvement of livestock has traditionally been based on phenotypic and pedigree information. Advances in molecular DNA technologies offer the opportunity to increase the rate of genetic gain using genetic markers (e.g., single-nucleotide polymorphisms, SNPs) [1,2]. For several species, including sheep, panels of more than 50,000 SNPs are currently available [3]. This technology enables the identification of genes or chromosomal segments that are associated with the traits of interest (Wenome-Wide Association Studies, GWAS) [4]. A number of statistical methods, including the single-step Bayesian regression approach, which combines all available pedigrees and phenotypic and genomic data, have been employed to conduct GWAS [4,5,6,7] and have been used for a number of livestock species.

In sheep, GWAS analyses have been performed for economically relevant traits such as the fiber diameter (FD), clean fleece weight (CFW), live weight (LW) and reproduction [8,9,10]. These traits are influenced by many genes, each with a small effect, and involve various cell types and tissues [11,12,13]. Nevertheless, candidate genes associated with major wool traits (FD and CFW) have been reported in the Australian and Chinese Merino sheep populations [8,13,14,15]. Genomic regions related to live weight have also been found in Merino sheep in Australia [9] and New Zealand [16]. A French study reported candidate genes associated with ewes’ body condition score (BCS) [17].

In Uruguay, few GWAS of livestock species have been undertaken or published [18,19,20]. There are no published GWAS of the wool, growth, or reproduction traits of Uruguayan sheep. The aim of this study was to detect the genomic regions and genes associated with the FD, CFW, LW, BCS and reproduction traits of Uruguayan ultrafine Merino ewes.

## 2. Materials and Methods

### 2.1. Ethical Statement

All animal work was approved by the INIA Animal Ethics Committee (INIA_2018.2).

### 2.2. Phenotypic and Pedigree Data

The data were derived from a Uruguayan Merino nucleus flock involved in a genetic program, as described by Ramos et al. [21,22]. The selection objectives, nutritional conditions and management of this flock were previously reported by Ramos et al. [22]. Phenotypic records of these six traits were obtained from approximately 2000 mixed-age ewes born between 1999 and 2018. The traits evaluated were the adult fiber diameter and clean fleece weight at late-pregnancy shearing (A_FD and A_CFW, respectively); live weight and body condition score at mating (LWM and BCSM, respectively); pregnancy rate (PR: pregnant or non-pregnant); and lambing potential (LP: the number of ultrasound-scanned fetuses per ewe combined: 0, 1 or ≥2). Details of the trait measurements were described by Ramos et al. [21,22]. The complete pedigree included 7168 animals.

### 2.3. Genotyping and Quality Control

Genomic DNA extraction from the blood samples was performed as described by Carracelas et al. [23]. The animals were genotyped using the GeneSeek^®^ Genomic Profiler™ Ovine 50K panel. Quality control was performed to remove SNPs with a minor allele frequency (MAF) lower than 1% and call rate below 85%, as well as animals with a call rate lower than 90%. After applying the quality control measures, 1133 animals and 40,036 SNPs were retained and utilized in the analyses.

### 2.4. Genome-Wide Association Study

Genome-Wide Association Studies were performed utilizing single-step Bayesian regression analyses implemented in the JWAS package [7]. The software tool JWAS is an open-source package for single-trait and multiple-trait genome-enabled prediction and analysis. JWAS is a single-language software that is easy for community members to use. The documentation and examples of JWAS can be found at https://reworkhow.github.io/JWAS.jl/latest/theory/theory accessed on 1 July 2022.

A Bayes C linear mixed model that included the genotyped and non-genotyped ewes was constructed. The model equation for the single-step Bayesian GWAS was as follows for the genotyped animals:

y = Xβ + Zu + Wpe + Mα + e(1)

where:

y = vector of phenotypes for the genotyped individuals,
β = vector of fixed effects,
u = vector of random animal genetic effects not explained by the markers,
pe = vector of random permanent environmental effects accounting for the covariance between observations of the same individual,
α = vector of marker effects,
e = vector of random residual effects,
X, Z and W = incidence matrices relating records to fixed, animal and permanent environmental effects,
M = genotype covariate (each coded as 0, 1 or 2).

The model equation for the non-genotyped individuals can be written as:

y = Xβ + Zu + Wpe + Mα + Z_n_ϵ + e (2)

where:

y = vector of phenotypes for the non-genotyped individuals,
M = genotype covariate matrix for the non-genotyped individuals imputed from the genotyped relatives,
Z_n_ = incidence matrix corresponding to the imputation residual,
ϵ = vector of imputation residuals accounting for errors in the genotype imputation.

All the other terms are as described in Equation (1).

A Markov chain Monte Carlo (MCMC) method was utilized to obtain samples from the posterior distributions of all unknown parameters, including the marker effects. A total of 70000 iterations were run after a burn-in of 5000 cycles and a sampling interval each 10 interactions. The probability that the markers would have null effect was set to 99% (parameter π = 0.99), that is, 1% of the 40036 SNPs (approximately 400 SNPs) was assumed to contribute to the genetic variance.

### 2.5. Detection of Important Windows Associated with the Trait and Candidate Genes

The genome was partitioned into 2015 non-overlapping windows of 20 consecutive SNPs which, on average, represented 1 Mb. Assuming that all the windows explained the same amount of variation, the expected proportion of genetic variance explained (PVE) by each window was 0.05% ([1/2015] ∗ 100). The 1 Mb windows that explained at least 0.25% of the genetic variance, which was 5 times the expected proportion of variance (0.05 × 5 = 0.25%), were considered to be the most important regions associated with the trait [24,25]. The top 10 windows for each trait that explained the largest PVE were examined to identify candidate genes. The annotated genes in those regions were extracted from the OAR v3.1 Ovine (Texel) Genome Assembly, available in the Ensembl database (http://www.ensembl.org/Biomart accessed on 10 August 2022) [26]. The biological functions of the genes were identified using the functional annotation tools in DAVID (https://david.ncifcrf.gov/tools.jsp accessed on 10 August 2022) [27]. Gene ontology (GO) enrichment analysis was conducted using g:Profiler (https://biit.cs.ut.ee/gprofiler/gost accessed on 1 September 2022) [28]. Pathways with a *p*-value < 0.05 were considered significantly enriched.

## 3. Results

### 3.1. Descriptive Statistics

A summary of the phenotypic records of the traits analyzed is shown in Table 1. The number of records ranged from 6288 to 7079.

### 3.2. Genome-Wide Association Study (GWAS)

**Figure 1:**
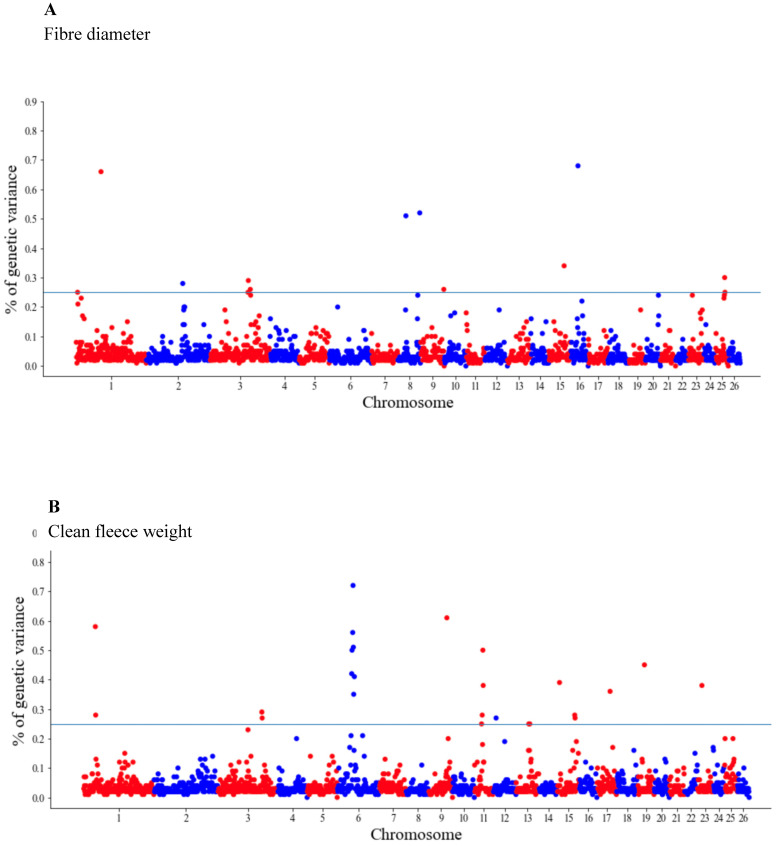
Manhattan plots of the genetic variance explained (%) by 20 adjacent SNP windows for the ewe fiber diameter (**A**) and clean fleece weight (**B**). Each dot represents a window. The % of additive genetic variance explained by each window and chromosomes 1–26 are shown on the *Y*-axis and *X*-axis, respectively. The horizontal line indicates the suggestive threshold of 0.25% of the PVE.

**Figure 2:**
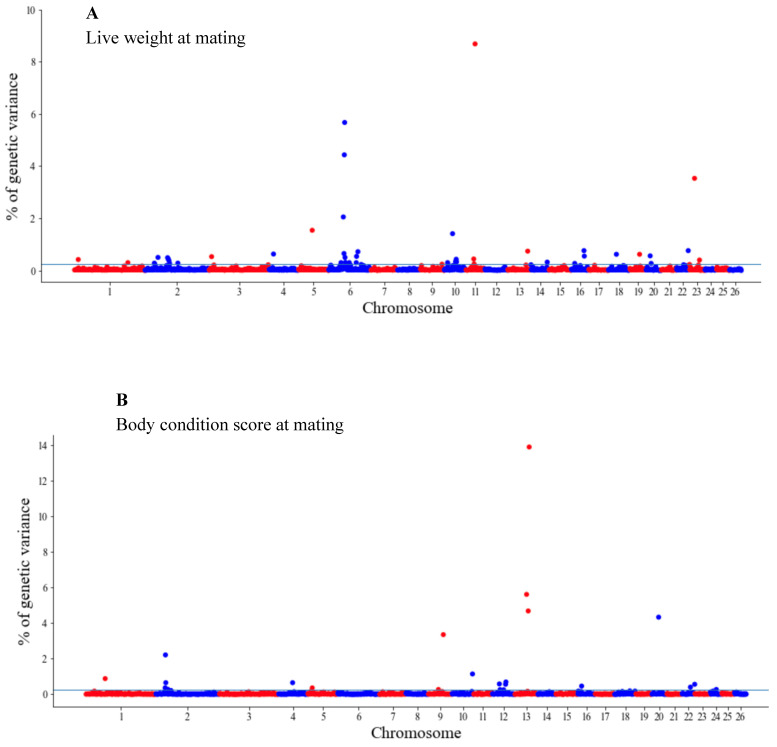
Manhattan plots of the genetic variance explained (%) by 20 adjacent SNP windows for the ewe live weight (**A**) and body condition score at mating (**B**). Each dot represents a window. The % of additive genetic variance explained by each window and chromosomes 1–26 are shown on the *Y*-axis and *X*-axis, respectively. The horizontal line indicates the suggestive threshold of 0.25% of the PVE.

**Figure 3:**
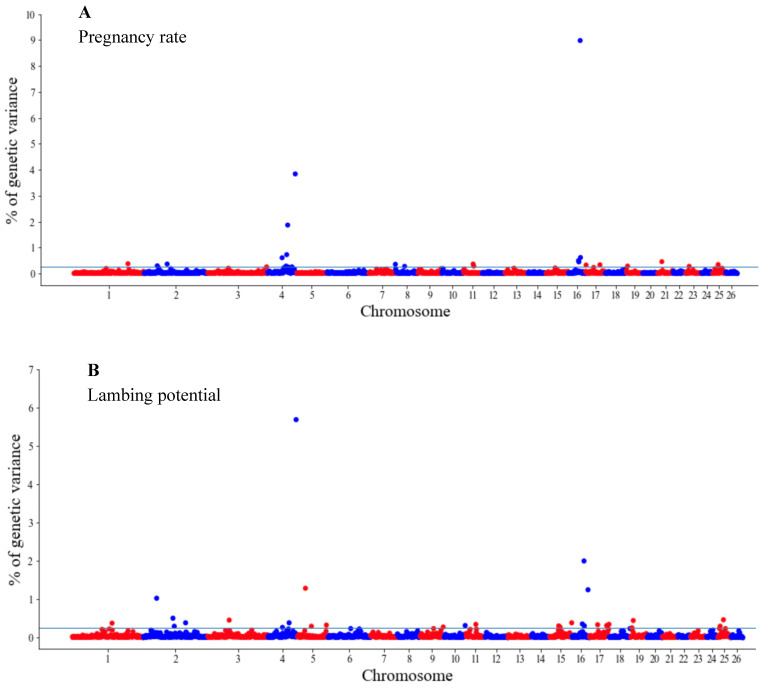
Manhattan plots of genetic variance explained (%) by 20 adjacent SNP windows for the pregnancy rate (**A**) and lambing potential (**B**). Each dot represents a window. The % of additive genetic variance explained by each window and chromosomes 1–26 are shown on the *Y*-axis and *X*-axis, respectively. The horizontal line indicates the suggestive threshold of 0.25% of the PVE.

The GWAS results are presented as the proportion of additive genetic variation explained by the windows of 20 consecutive SNPs, as reported by other authors [13]. Manhattan plots illustrating the proportion of additive genetic variation explained by each window of 20 adjacent SNPs for the wool, body and reproduction traits are presented in Figure 1, Figure 2 and Figure 3, respectively. The suggestive threshold of 0.25% of the PVE is indicated by the horizonal blue line.

For the wool traits, a total of 35 windows (13 for FD and 22 for CFW) surpassed the significance threshold (PVE ≥ 0.25%, Figure 1). The proportion of the total additive genetic variance explained by these windows was 4.85 and 9.06% for FD and CFW, respectively. There were 42 windows significantly associated with LWM, which collectively explained 43.2% of the additive genetic variance. For BCS, 22 relevant windows accounted for more than 40% of the additive genetic variance (Figure 2). For the reproduction traits, 53 genomic windows (24 and 29 for PR and LP, respectively) reached the suggestive threshold of 0.25% of the PVE (Figure 3).

### 3.3. Top 10 Genomic Regions and Candidate Genes

The chromosome, location, PVE and candidate genes within the top 10 windows for each trait are shown in Table 2, Table 3 and Table 4. The top 10 windows cumulatively explained 4.1, 5.3, 29.6, 37.4, 18.4 and 13.5% of the additive genetic variance for the A_FD, A_CFW, LWM, BCSM, PR and LP, respectively. Some of these windows were associated with more than one trait. For example, three genomic regions on chromosome 6 were associated with both the CFW and LWM. Similarly, two overlapping regions were associated with the PR and LP. A total of 240 genes were contained within the top 10 genomic regions across the six traits.

### 3.4. Enrichment Analysis

A gene ontology (GO) enrichment analysis of the genes within the top 10 windows for each trait was performed. GO terms with a *p*-value < 0.05 were considered significantly enriched. The enriched terms were associated with molecular functions (MF), biological processes (BP) and/or cellular components (CC). In total, 20 GO terms were enriched (*p*-value < 0.05). More information from the GO analysis is available in the Supplementary Materials.

## 4. Discussion

The present study reports on chromosome segments associated with economically relevant traits of Uruguayan Merino sheep. The genomic regions of interest on chromosomes 1, 3, 4, 5, 6, 8, 9, 11, 12, 13, 16, 19 and 22 identified in this study contain known candidate genes related to the wool traits, live weight, body condition score and reproduction traits of sheep and other species (see the following paragraphs). This section focused on some of the genes located within the top 10 windows for each trait that explained the largest proportion of the additive genetic variance.

Wool follicle regulation involves several genes, including IGF and TGFB [29]. The candidate genes for the wool traits identified in this study included IGF-1, TGFBR2, PDE3A, STK3, PRKCA, ZNF704 and EXT1. These genes have previously been associated with hair [30,31,32], cashmere [33,34] and wool [35,36,37]. In our study, the functional analysis indicated that the genes WAPL, ESR1 and IGF-1 were significantly enriched in the regulation of fibroblast proliferation (see Supplementary Materials), which is crucial for hair follicle formation [38]. Overall, the proportion of additive genetic variance explained by each window was relatively small (lower than 0.75%), which reflects the polygenic nature of wool traits.

The identification of genes associated with the live weight is of particular interest for sheep breeding programs [13,39]. Some of the potential genes for the ewe LWM are known to be involved in the LW of young sheep. For example, CAST has been related to the birth weight and growth rate of lambs [40,41]. The genes LAP3, MED28, and HERC6, located on chromosome 6, have previously been identified as candidate genes for the post-weaning LW in Australian Merino sheep [9]. The identification of genes commonly affecting the LW at both early and adult ages is unsurprising, given the moderate to high genetic correlations between these traits [42,43]. The genes HERC6 and MED28 have been also associated with gastrointestinal nematode infection, which is one of the most important health problems in grazing sheep [44]. This is in agreement with earlier studies that suggested that some SNPs associated with gastrointestinal nematode resistance are involved in growth traits [45]. In sheep, the gene GPRIN3 was also reported to be associated with the litter size [46]. Other genes that were found to be associated with the ewe LWM in the present study have been reported as candidate genes for LW-related traits in different species. For example, A1CF, ZNF830, CCT6B and MYO10 were associated with residual feed intake in cattle [47,48,49], while the genes CTBP2 and AP2B1 have been linked to meat quality and lipid metabolism in pigs [50,51].

In sheep, the body condition score is an indicator of the available body reserves (fat and muscle) [52]. Several genes that are known to be involved in fat storage and metabolism were associated with the ewe BCS in the present study. For example, TMC2, SIRPA and CPXM1 have been reported as candidate genes for tail fat deposition in sheep [53]. The gene CPXM1 was also identified as a positive regulator of adipogenesis in mice and humans [54]. In mice, FMO1, a member of the flavin-containing mono-oxygenase (FMO) gene family, was associated with energy homeostasis and metabolic efficiency [55]. TPD52 is a regulator of lipid metabolism and is involved in fatty acid storage [56]. Early studies indicated that the overexpression of ATG3 favors lipid deposition in mice [57]. In humans, LRRC1 is involved in adipocytic differentiation [58], whereas F13A1 is expressed at high levels in the adipose tissue of obese individuals [59].

The present study identified two common regions associated with pregnancy and the lambing potential, suggesting that the same genes may play a role in the regulation of these reproduction traits. These regions are located on chromosomes 4 and 16 and contain genes that have previously been associated with several reproduction traits. For example, ADCY1 has been related to pubertal initiation in sheep [60], fertility in dairy cattle [61] and fecundity in goats [62,63]. The gene CDH10 was associated with several reproduction traits in buffaloes [64]. Furthermore, PDIA4 was found to be involved in the litter size in sheep and pigs [65,66]. In our work, LEPR was identified as a candidate gene for the pregnancy rate. This gene has already been associated with several reproduction traits, including pregnancy [67,68,69,70]. The genes GHR and LPAR2 are associated with sheep reproduction [71,72], while DMXL1 is related to reproduction traits in heifers [73]. Therefore, there is evidence supporting the concept that the genes located on chromosomes 4 and 16 play an important role in the reproduction traits of Merino sheep.

This work was the first to perform a single-step Genome-Wide Association Study of the production and reproduction traits of mixed-age ewes in Uruguay. As mentioned above, several of the candidate genes detected were also reported in other studies, which provides confidence in our results. A limitation of this study was the small sample size, which affected the power of detection. Future analyses based on larger populations would improve the identification of candidate genes for traits of interest in sheep. Our results will contribute to the Uruguayan Merino genetic evaluation, as some of identified genes are good targets for selection. In addition, the genomic regions identified here should be utilized as targets in further studies.

## 5. Conclusions

This study performed a single-step GWAS of six traits in a Uruguayan Merino sheep population. A total of 13, 22, 42, 22, 24 and 29 genomic regions were significantly associated with the fiber diameter, clean fleece weight, live weight at mating, body condition score at mating, pregnancy rate and lambing potential, respectively. We detected several genes, some of which were novel, showing potential associations with the wool (IGF-1, TGFB2R, PRKCA), live weight (CAST, LAP3, MED28, HERC6), body condition score (CDH10, TMC2, SIRPA, CPXM1) or reproduction traits (ADCY1, LEPR, GHR, LPAR2) of mixed-age ewes. These results require validation using a larger dataset before their implementation in genomic selection among Uruguayan Merino sheep. Overall, our findings will be useful for further genomic studies and genetic improvement programs in Uruguay.

## Figures and Tables

**Table 1:** Descriptive statistics for the wool, body growth and reproduction traits of mixed-age ewes born between 1999 and 2018.

Trait	Mean	SD ^1^	Records
Fibre diameter (A_FD, μm)	16.6	1.75	7079
Clean fleece weight (A_CFW, kg)	2.80	0.51	6288
Live weight at mating (LWM, kg)	47.4	5.97	6589
Body condition score at mating (BCSM)	3.2	0.65	6442
Pregnancy rate (PR)	0.73	0.44	6376
Lambing potential (LP)	0.91	0.66	6376

^1^ SD: standard deviation of the raw phenotypic records.

**Table 2:** Chromosome, location, proportion of additive genetic variance (PVE, %) and candidate genes within the top 10 windows associated with the fiber diameter (A_FD) and clean fleece weight (A_CFW) of Merino ewes.

Trait	Chr	Window Bounds (bp)	PVE (%)	Candidate Genes
A_FD	1	221,133,506–241,462,240	0.66	-
	2	132,440,576–134,761,891	0.28	*HOXD10, OLA1, SP9, CHN1, CHRNA1, MTX2*
	3	171,178,810–174,258,094	0.29	*IGF-1, PAH, STAB2, NT5DC3, GLT8D2, SLC41A2, TDG*
	3	191,554,038–194,804,430	0.26	*PDE3A, C2CD5, ST8SIA1, HPCAL1, KCNJ8, PYROXD1, SLCO1C1*
	8	35,247,461–84,539,654	0.52	*ESR1, PLEKHG1, NT5E, NHSL1, ANKRD6, CGA, PLEKHG1*
	8	66,612,859–69,354,718	0.51	*ADGRG6, PHACTR2, UTRN, VTA1*
	9	48,994,813–16,528,100	0.26	*PRDM14, WWP1, EXT1, MATN2, PTDSS1, ZNF704*
	15	47,505,272–4,779,161	0.34	*DYNC2H1, LOC101106199, LOC101105437*
	16	52,688,043–60,285,005	0.68	*ARHGAP22, CDH18, TSNAX*
	25	36,631,465–40,822,020	0.30	*WAPL, GRID1*
A_CFW	1	111,294,404–122,825,051	0.58	*UHMK1, DDR2, NUF2, ATF6, INPP5B, RGS5*
	6	36,295,216–36,872,516	0.72	*BBS7, HERC6, CCNA2, LOC101120495,*
	6	36,066,911–36,286,475	0.56	*HERC3, HERC5, HERC6, PYURF, PIGY*
	6	36,905,457–37,129,550	0.51	*LAP3, MED28,*
	6	35,191,867–35,728,962	0.50	*GPRIN3, TIGD2*
	6	33,844,752–35,184,703	0.42	*MMRN1, CCSER1,*
	6	37,767,491–38,052,441	0.41	*-*
	9	77,283,695–85,378,072	0.61	*STK3, MTDH, MATN2, OSR2, VPS13B*
	11	66,432,553–10,722,809	0.50	*PRKCA, DHX40, LOC101102402, COIL, INTS2, PPM1E, SRSF1*
	19	4,811,675–54,605,752	0.45	*PBRM1, TGFBR2, BAC5, RBM6, CACNA2D3, DCP1A, MAP4*

Chr, chromosome; PVE (%), proportion of additive genetic variance explained by each window; A_FD, adult fiber diameter; A_CFW, adult clean fleece weight, bp: base pairs.

**Table 3:** Chromosome, location, proportion of additive genetic variance (PVE, %) and candidate genes within the top 10 windows associated with the live weight (LWM) and body condition score (BCSM) at mating of Merino ewes.

Trait	Chr	Window Bounds	PVE (%)	Candidate Genes
LWM	5	93,416,569–93,461,942	1.54	*CAST*
	6	36,905,457–37,129,550	5.67	*LAP3, MED28*
	6	36,295,216–36,872,516	4.43	*BBS7, HERC6, CCNA2, LOC101120495*
	6	35,191,867–35,728,962	2.05	*GPRIN3, TIGD2*
	10	57,396,545–59,143,370	1.41	*-*
	11	13,795,276–16,306,848	8.68	*LOC101110777, AP2B1, CCT6B, ZNF830*
	13	85,702,745–58,406,417	0.74	*GPR158, MKX, SYNDIG1, PREX1*
	16	54,796,169–58,047,574	0.76	*MYO10, CPEB4*
	22	55,395,817–44,154,558	0.76	*A1CF, CTBP2, GRK5, XPNPEP1, CFAP46, DOCK1, INSYN2A, LIPA, MUOF, PLCE1*
	23	36,106,915–38,820,448	3.53	*MYOM1, DLGAP1, SMCHD1*
BCSM	1	173,862,929–190,851,636	0.88	*ATP6V1A, CD200, ATG3, CFAP44, CCDC191, NECTIN3, NEPRO, PLCXD2, SLC9C1*
	2	111,201,892–114,207,253	2.21	*HPGD, ECPAS, FBXO8, GLRA3*
	2	114,746,188–116,378,689	0.65	*GALNTL6*
	9	57,388,779–56,572,469	3.35	*STMN2, TPD52, ZBTB10*
	10	56,843,164–69,129,301	1.14	*LOC101115632, SPATA13*
	12	36,958,022–40,040,678	0.69	*FMO1, FMO2, FMO4, MTHFR, MFN2, PRRC2C, TNFRF1B*
	13	51,269,879–54,158,422	13.89	*TMC2, SIRPA, CPXM1, KCNQ2, RBBP8NL, DNAAF9*
	13	26,807,364–30,712,871	5.61	*ITGA8, FRMD4A, MINDY3, RSU1, ANKEF1, CUBN, FAM171A1, PTER, PRPF18, TRDMT1*
	13	42,377,205–45,741,876	4.68	*PGF2, LOC106990122, LOC101108592*
	20	52,019,528–6,887,963	4.33	*KHDRBS2, F13A1, GMDS, CDYL, HCRTR2, LRRC1, LOC101114063*

Chr, chromosome; PVE (%), proportion of additive genetic variance explained by each window; LWM and BCS, live weight and body condition score at mating, respectively.

**Table 4:** Chromosome, location, proportion of additive genetic variance (PVE, %) and candidate genes within the top 10 windows associated with the pregnancy rate (PR) and lambing potential (LP) of Merino ewes.

Trait	Chr	Window Bounds	PVE (%)	Candidate Genes
PR	1	40,646,909–41,530,079	0.38	*LEPR, DNAJC6*
	4	107,666,587–70,951,540	3.84	*ADCY1, PDIA4, LOC101113583, LRRC4, SND1, TAC1*
	4	52,544,546–58,541,568	1.87	*-*
	4	42,940,192–48,319,572	0.73	*NAPEPLD, PTPN12, RELN, FBXL13, GSAP, FAM185A, ORC5*
	4	113,244,910–125,545,756	0.61	*RBM33, XRCC2, CNPY1, LMBR1, PPP1R9A, RNF32*
	16	45,966,691–50,076,565	8.98	*CDH10*
	16	50,280,874–54,676,335	0.62	*CDH12*
	16	25,883,094–30,276,428	0.50	*PARP8*
	16	30,392,117–33,873,022	0.45	*OXCT1, CARD6, GHR, CCL28, RIMOC1, RANBP17, MROH2B, PAIP1*
	21	18,437,167–23,229,347	0.46	*FAT3, LOC101117547, LUZP2*
LP	2	138,951,962–150,539,090	1.03	*LRP2, CERS6, STK39*
	2	67,932,661–69,907,932	0.51	*ABHD17B, GDA, TRPM3*
	2	195,395,266–197,769,393	0.39	*HECW2, DNAH7, SLC39A10*
	3	192,191,853–194,520,670	0.46	*PDE3A, LOC101117577, C2CD5, SLCO1A2, SPX, LOC101115359*
	4	107,666,587–70,951,540	5.68	*ADCY1, PDIA4, LOC101113583, LRRC4, SND1, TAC1*
	5	30,712,220–3,475,920	1.29	*LPAR2, DMXL1, FLT4,*
	16	45,966,691–50,076,565	2.00	*CDH10*
	16	1,653,965–4,370,649	1.25	*DOCK2, MROH2B, BDP1, LOC101122306, ANKRD55, SLIT3, SGTB*
	19	60,339,892–10,882,333	0.45	*LRRFIP2, STAC, TRANK1*
	25	38,239,943–41,537,781	0.47	*CCSER2*

Chr, chromosome; PVE (%), expected proportion of additive genetic variance explained by each window; PR, pregnancy rate; LP, lambing potential.

## Data Availability

The data presented in this study are available within the article.

## Supplementary Materials

The following supporting information can be downloaded at: https://www.mdpi.com/article/10.3390/genes14010167/s1, Table S1: Enrichment analysis.
