# Supplementary material for: Genomic Regions Associated with Wool, Growth and Reproduction Traits in Uruguayan Merino Sheep
Source: Genes (Basel). 2023 Jan 7;14(1):167. doi: 10.3390/genes14010167 (PMC9858812; doi:10.3390/genes14010167)
Supplement: Supplementary file 1 [file genes-14-00167-s001.zip › genes-2128229-supplementary.pdf]

Table S1. Significant gene ontology terms associated with biological processes (BP), molecular functions (MF), and cellular components (CC) associated with fibre diameter (A), clean fleece weight (B), body condition score (C), pregnancy rate (D) and lambing potential (E).

A: Fibre diameter

GO category	GO term	adjusted_p_value	Gene names
**MF**	oxygen carrier activity	3.936×10^{-3}	LOC101106199, LOC101105437
	oxygen binding	2.539×10^{-2}	LOC101106199, LOC101105437
**BP**	regulation of oocyte development	6.077×10^{-3}	IGF-1, PDE3A
	regulation of oogenesis	6.077×10^{-3}	IGF-1, PDE3A
	positive regulation of fibroblast proliferation	1.372×10^{-2}	WAPL, ESR1, IGF-1
	oxygen transport	4.163×10^{-2}	LOC101106199, LOC101105437
	regulation of fibroblast proliferation	4.883×10^{-2}	WAPL, ESR1, IGF-1
	gas transport	4.994×10^{-2}	LOC101106199, LOC101105437
**CC**	hemoglobin complex	2.988×10^{-3}	LOC101106199, LOC101105437

B: Clean fleece weight

GO category	GO term	adjusted_p_value	Gene names
**MF**	protein kinase activity	1.869×10^{-2}	UHMK1,DDR2, STK3, LOC101102402, PRKCA
**MF**	phosphotransferase activity, alcohol group as acceptor	3.972×10^{-2}	UHMK1, DDR2, STK3, LOC101102402, PRKCA
**CC**	neuronal ribonucleoprotein granule	2.143×10^{-2}	UHMK1

C: Body condition score

GO category	GO term	adjusted_p_value	Gene names
**GO:MF**	N,N-dimethylaniline monooxygenase activity	5.740×10^{-4}	FMO1, FMO2, FMO3
	NADP binding	3.879×10^{-3}	GMDS, FMO1, FMO2, FMO3
	oxidoreductase activity, acting on paired donors	3.175×10^{-2}	FMO1, FMO2, FMO3

D: Pregnancy rate

GO category	GO term	adjusted_p_value	Gene names
**GO:BP**	postsynaptic membrane organization	1.002 x 10^{-2}	LRRC4,ENSOARG00000017524, Membrane associated guanylate kinase, WW and PDZ domain containing 2, RELN
**GO:BP**	postsynaptic density protein 95 clustering	2.604 x 10^{-2}	LRRC4, RELN
**GO:CC**	integral component of postsynaptic density membrane	2.945 x 10^{-2}	ADCY1, LRRC4
**GO:CC**	intrinsic component of postsynaptic density membrane	3.577 x 10^{-2}	ADCY1, LRRC4

E: Lambing potential

GO category	GO term	adjusted_p_value	Gene names
**GO:BP**	transmembrane transport	1.749 x 10^{-2}	LRP2, STK39, TRPM3, SLC39A10, LOC101117577, C2CD5, SLCO1A2

BP: Biological processes; **MF**: Molecular functions; **CC**: Cellular components.
